# Intravenous zoledronate for pediatric langerhans cell histiocytosis with bone involvement

**DOI:** 10.3389/fped.2026.1825076

**Published:** 2026-04-29

**Authors:** Jann Adriel Sy, Thuan Chong Quah, Pian Pian Tee, Cindy Wei Li Ho

**Affiliations:** 1 Department of Paediatrics, Yong Loo Lin School of Medicine, National University of Singapore, Singapore, Singapore; 2 Khoo Teck Puat - National University Children’s Medical Institute, National University Health System, Singapore, Singapore; 3 Department of Paediatrics, Sabah Women and Children Hospital, Sabah, Malaysia

**Keywords:** bisphosphonate therapy, Langerhans cell histiocytosis, pediatric bone lesions, skeletal relapse, zoledronate

## Abstract

Bone involvement in pediatric Langerhans cell histiocytosis (LCH) causes pain, functional impairment, and frequent relapse, creating a need for adjunctive therapies with rapid skeletal benefit. At National University Hospital, Singapore, a tertiary pediatric hematology-oncology referral centre, we retrospectively evaluated eight children with osseous LCH treated with intravenous zoledronate between January 2020 and December 2025. Three had multisystem disease (two risk-organ–positive), four had multifocal bone LCH, and one had unifocal bone LCH. Pain response (clinical improvement) and radiologic outcome (lesion stability or sclerosis) and adverse effects, and concurrent therapies were recorded. Prior treatment exposure was heterogeneous, ranging from no prior systemic therapy to multi-agent chemotherapy, oral maintenance therapy, targeted therapy, radiotherapy, pamidronate, and indomethacin. Zoledronate was given for painful active bone lesions, particularly in weight-bearing sites, and in selected patients for progressive relapsed osseous disease, poor tolerance of oral chemotherapy, or as a chemotherapy-sparing bone-directed approach. Zoledronate was started at a median age of 60 months. All 4 symptomatic patients experienced pain improvement (100%), with a median time to improvement of 18 days post-infusion. Among 8 patients with imaging follow-up, 2 exhibited complete radiographic resolution and 6 showed improvement. The treatment was well tolerated; transient fever occurred in 4 patients. Three patients received concomitant oral chemotherapy for active systemic disease, either in other systems or in new bone lesions. No cases of clinically significant hypocalcemia or osteonecrosis of the jaw were observed.

## Introduction

Langerhans cell histiocytosis (LCH) is a rare disorder characterized by clonal proliferation of dendritic cells that may infiltrate bone, skin, liver, lungs, and other organs (1). Bone involvement is the most common manifestation and typically presents with pain, swelling, or pathologic fractures. Bone is also one of the most frequent sites of relapse (2).

Systemic chemotherapy remains the standard of care for multisystem LCH, guided by protocols such as the Histiocyte Society LCH-III trial (3). However, isolated or recurrent bone lesions may persist despite frontline or salvage therapy. Lesions in weight-bearing bones or the spine may require faster-acting agents to achieve rapid symptom control and prevent functional compromise. These challenges underscore the need for effective adjuvant strategies targeting osseous disease.

Bisphosphonates inhibit osteoclast-mediated bone resorption by inducing osteoclast apoptosis (4). Zoledronate, a third-generation nitrogen-containing bisphosphonate, is several hundred-fold more potent than earlier agents (5). In pediatric practice, intravenous zoledronate has demonstrated efficacy in osteogenesis imperfecta, glucocorticoid-induced osteoporosis, fibrous dysplasia, and malignancy-associated hypercalcemia (6).

Bisphosphonate use in LCH was first reported in 1989, when clodronate was associated with pain relief in multifocal eosinophilic granuloma (7). Since then, case reports have described the successful use of newer-generation bisphosphonates, such as zoledronate, in both adult and pediatric LCH cases, although data in children remain lacking, especially with the use of zoledronate (8).

We report our institutional experience with intravenous zoledronate in pediatric bone LCH, focusing on clinical response, radiologic outcomes, safety, and potential therapeutic indications.

## Materials and methods

We conducted a retrospective review of pediatric patients with LCH treated with intravenous zoledronate at the National University Hospital, Singapore, between 2020 and 2025. Patients were included if they received at least one dose of zoledronate for LCH. The study was approved by the Institutional Review Board (Domain Specific Review Board, National Healthcare Group, Singapore).

Demographic and clinical variables included age at diagnosis and treatment, disease classification at diagnosis (unifocal vs. multisystem), presenting symptoms, number and interval of zoledronate doses, pain and imaging responses, concurrent therapies, and adverse events. Radiographic responses were based on follow-up x-rays or written reports.

Primary outcomes in the analysis included clinical response and radiological improvement. Clinical response was defined as subjective improvement or resolution of pain and/or limping in the lower limbs. Complete radiological response was defined as the complete resolution of the lytic lesion. Improvement was defined as a decrease in size and/or number of lesions. Finally, a mixed response was defined as the resolution or improvement of one or more lesions, with the development of new lesions.

## Results

### Patient characteristics

Eight patients received intravenous zoledronate, administered at 3–4-month intervals (1–4 doses per patient). Three had multisystem LCH (two with risk-organ involvement), four had multifocal bone disease, and one had unifocal bone LCH. The median age at diagnosis was 20 months, and the median age at initiation of zoledronate was 60 months. Notably, zoledronate was not given in the setting of clear overlap with effective systemic chemotherapy. At the time of zoledronate initiation, patients either had no prior systemic treatment, had completed prior chemotherapy months earlier, or were experiencing symptomatic relapse/progression despite oral therapy. This included patients whose last systemic chemotherapy had been 8–24 months earlier, as well as a patient whose skull lesion enlarged despite 2 months of prednisolone, 6-mercaptopurine and methotrexate. The main indications for zoledronate were painful active bone lesions, particularly in weight-bearing sites, progression of bony disease despite prior therapy, poor tolerance of oral chemotherapy, and the desire to provide a less intensive steroid-/chemotherapy-sparing approach in heavily pretreated or multiply relapsed disease. Table 1 summarizes the clinical findings of the eight patients who received IV zoledronate. Detailed clinical vignettes of the eight cases may be found in supplemental text 1, along with representative radiographs for some of the patients (Supplementary Figures S1–S4).

**Table 1:** Summary of patients treated with IV zoledronate for LCH.

Case	Age/sex at diagnosis of LCH	Race	Diagnosis	Age at first IV zoledronate	Bone lesions at commencement of therapy	Indication for IV Zoledronate	Date of last systemic treatment prior to zoledronate	Dose	# of infusion	Treatment phase	Vit D level (ug/L)	iCal level (mmol/L)	Side effects	Combined therapy	Reason for combined therapy	Radiological response	Clinical response	Time to Symptom Resolution after 1st dose
1	15 Months/Female	Malay	Multisystem LCH (Risk Organ+)	8 Years 3 months	Multiple bones—skull and tibia	Painful lesions, poor tolerance to oral chemotherapy	Ongoing oral 6MP and MTX 2 years	0.02 mg/kg—1st and 2nd 0.04 mg/kg—3rd	3	Relapsed	24.3	1.2	None	Vemurafenib, prednisone	Flare of LCH in other systems (non-bone)	Improvement	Resolution of pain	25 days
2	3 Years 11 months/male	Chi	Unifocal bone LCH	3 Years 11 months	Left tibia	Weight bearing bone and pain	No systemic chemotherapy	0.02 mg/kg	4	Frontline	23.9	1.17	Fever and emesis	None	N/A	Complete resolution	Resolution of pain	28 days
3	5 years 8 months/female	Chi	Multifocal bone LCH	5 Years 10 months	Left femur	Weight bearing bone and pain	No systemic chemotherapy	0.02 mg/kg	1	Frontline	37	1.08	Fever	6 mercaptopurine, prednisone	New scalp lesion 1 month after IV zoledronate dose	Complete resolution	Resolution of pain	11 days
4	14 Months/female	Viet	Multisystem LCH (Risk organ-)	4 Years 6 months	Multiple skull bones	New bone lesions over scalp. Previously heavily treated and limited finances	Ongoing 6MP and MTX for about 1 year	0.02 mg/kg	1	Relapsed	41	1.18	None	None	N/A	Improvement	Asymptomatic pre-infusion	N/A
5	13 Months/female	Viet	Multisystem LCH (Risk organ+)	3 Years 4 months	Left hip, left femur	New painful lesions over right cheek, left thigh, weight-bearing bone.	Systemic chemotherapy ended 11 months prior to zoledronate	0.02 mg/kg	3 (ongoing)	Relapsed	24	1.23	Fever	Yes—oral 6 mercaptopurine and methotrexate	New onset lesions over the proximal femur	After 1st dose—mixed response—improvement in previous lesion, new lesion in another part of left femur	Resolution of pain and limp	7 days
																After 2nd dose—Improvement sclerosis and smaller lesions		
6	24 Months/female	Viet	Multifocal bone LCH	5 Years	Right parietal bone, vertebra plana, left ischium, right third metacarpal, left medial proximal femur	Lesions in weight bearing bone—spine although asymptomatic.	Systemic chemo ended 8 months prior to zoledronate	0.02–0.025 mg/kg	2 (ongoing)	Relapsed	76	1.27	Fever	None	N/A	Improvement	Asymptomatic pre-infusion	N/A
																Lytic lesion in parietal bone resolved. Interval rim sclerosis in lesions of left ischium and left medial proximal femur. Rest of previous lesions stable		
7	24 Months/male	Viet	Multifocal bone LCH	3 Years 3 months	Skull bone	Pain over left parietal region, progressing despite prednisolone and 6MP MTX	Ongoing 6mp and MTX and prednisolone for 2 months prior to zoledronate	0.02–0.025 mg/kg	3 (ongoing)	Relapsed	35	1.21	None	None	N/A	After 1st infusion—mixed response—slightly larger lesions, but borders demonstrating sclerosis	Asymptomatic pre-infusion	N/A
																After 2nd dose—improvement with resolution of lesions and smaller lesions.		
8	20 Months/female	Jap	Multifocal bone LCH	13 Years	Skull lesions (new), right tibial shaft (old), left proximal tibia (new). T4, T5, T8, T12 Vertebrae (old)	Multiply relapsed, NSAID-sparing option	Oral indomethacin last given 1 month prior to presentation	0.025 mg/kg	2 (ongoing)	Relapsed	27	1.17	None	None	NA	Improvement	Asymptomatic pre-infusion	N/A
																Resolution of skull lesion, stability of old lesions		

### Clinical and radiologic outcomes

**Figure 1:**
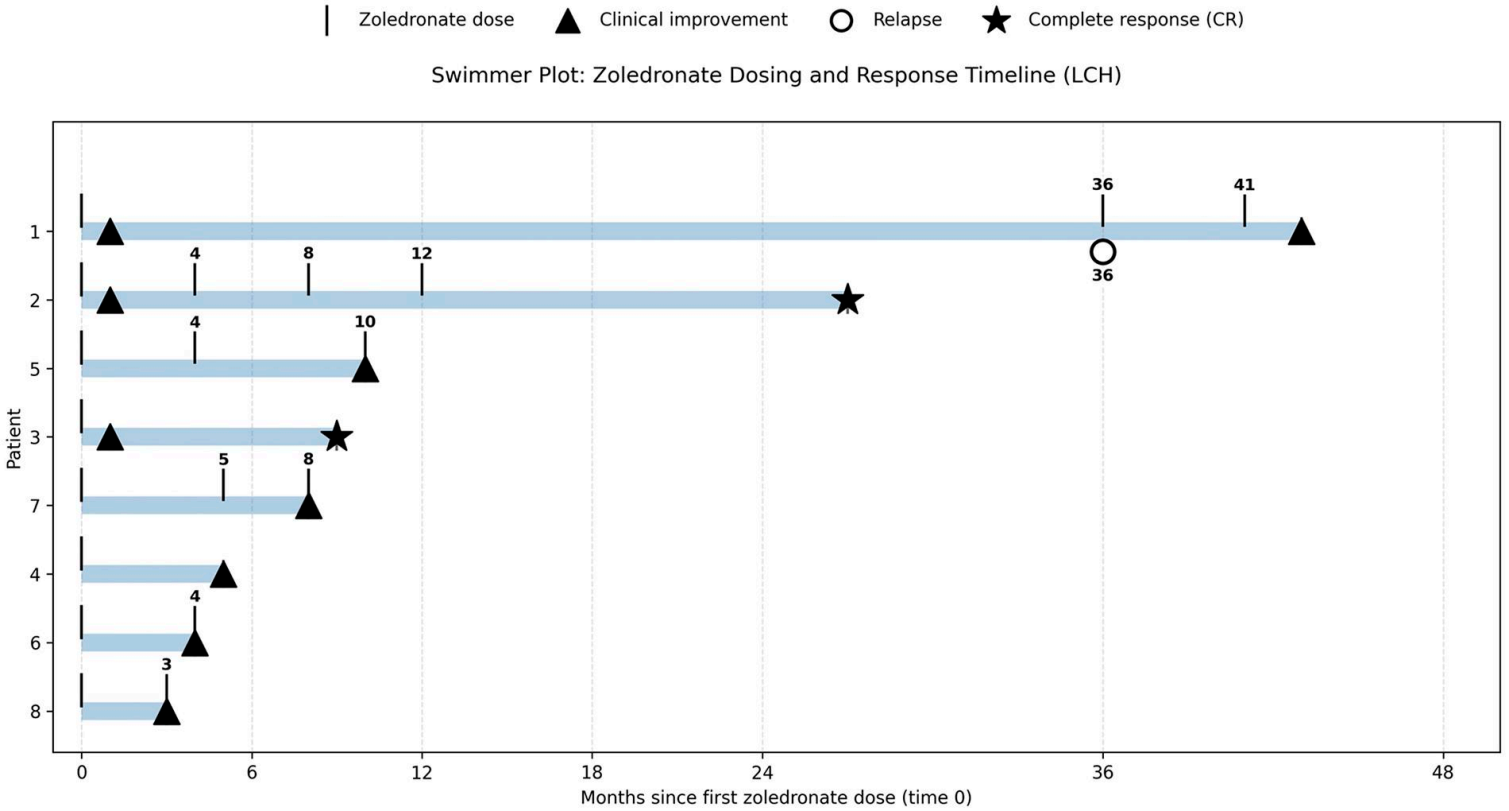
Zoledronate treatment timeline and response in pediatric bone LCH. Swimmer plot illustrating treatment course for eight children receiving intravenous zoledronate. Time is measured in months from first zoledronate administration (month 0). Vertical ticks represent subsequent doses. Triangles indicate clinical improvement, open circles relapse, and stars complete response (CR). Bar length represents follow-up duration from treatment initiation.

Pain improvement was observed in all 4 symptomatic patients, with a median resolution time of 18 days. Radiologic response was noted in all patients with 2 achieving complete resolution and 6 demonstrating radiologic improvement. The swimmer plot (Figure 1) illustrates treatment timing and response patterns. Most patients remain under follow-up and will receive additional doses as indicated. Three patients received concomitant systemic therapy due to ongoing disease activity: one received oral vemurafenib, one received oral methotrexate with 6-mercaptopurine, and one received 6-mercaptopurine alone.

### Safety

Zoledronate was well tolerated. Four patients developed transient fever following infusion. No cases of osteonecrosis of the jaw or symptomatic hypocalcemia were observed. Baseline vitamin D and calcium levels were confirmed to be adequate prior to treatment initiation.

## Discussion

Our series represents, to our knowledge, the largest single-center cohort of pediatric LCH patients treated with intravenous zoledronate to date. The most striking finding was the consistent local response observed across the cohort, with radiologic improvement seen in all patients and rapid pain relief in all symptomatic patients. Importantly, zoledronate was often used in clinical situations where its contribution could be meaningfully appreciated: several patients had no overlapping effective chemotherapy, had completed prior systemic therapy months earlier, or were already relapsing despite oral treatment at the time of zoledronate initiation. These findings support zoledronate as an active bone-directed therapy rather than merely a coincidental adjunct. In our cohort, its indications extended beyond analgesia alone. In some patients, treatment was clearly symptomatic, directed at painful active lesions causing limp, nocturnal pain, skull pain, or functional limitation, particularly in weight-bearing bones. In others, the indication was more preventive or structural, aiming to promote sclerosis and osseous healing, stabilize lesions in vulnerable skeletal sites such as the spine or lower limbs, and potentially reduce subsequent skeletal morbidity.

A targeted review of the published literature provides context for these findings. In a targeted PubMed search, we identified 9 studies describing bisphosphonate use in pediatric LCH, comprising 28 reported cases (8–16). Most involved relapsed or refractory disease. The majority (22/28) received intravenous pamidronate; only two cases involved zoledronate. Overall, improvement was reported in 23/28 cases (82%), with symptom resolution typically within 2–3 months. Reported toxicities were generally mild, and no osteonecrosis of the jaw was described. Supplementary Table S1 describes the cases collated into a table. Our findings therefore extend the existing literature in two important ways: first, by adding the largest focused experience with zoledronate in children with LCH; and second, by suggesting that zoledronate may achieve not only symptomatic relief but also consistent radiologic healing in selected patients with osseous disease.

A significant proportion of the existing literature focuses on pamidronate and its use has been described since the early 1990s and 2000s. Compared to pamidronate, zoledronate offers several practical advantages. It is a nitrogen-containing bisphosphonate that inhibits farnesyl pyrophosphate synthase in the osteoclast mevalonate pathway and has vastly higher potency and a prolonged skeletal half-life compared to pamidronate and previous generation bisphosphonates (6). It can be administered every 3–4 months with a short infusion time (approximately 30 min), compared to 3–4 h every month for pamidronate. This reduces treatment burden and improves feasibility in outpatient and resource-limited settings. Zoledronate may be particularly useful in multifocal bone disease or lesions affecting weight-bearing bones such as the femur or spine. Rapid pain relief may facilitate earlier mobilization, reduce reliance on corticosteroids or opioids, and potentially prevent orthopedic complications.

Beyond anti-resorptive effects, bisphosphonates may exert anti-inflammatory and immunomodulatory effects. LCH lesions contain osteoclast-like giant cells expressing TRAP, cathepsin K, and MMP-9, driven by RANKL and M-CSF produced by LCH cells and lesional T cells (17). Bisphosphonates may modulate this inflammatory microenvironment. Reductions in inflammatory cytokines (e.g., IL-6, IL-1, TNF-α) have been reported in patients treated with pamidronate (18).

Zoledronate may also be particularly advantageous in low- and middle-income countries, where infrequent dosing and lack of myelosuppression make it a practical adjunct to systemic therapy. Unlike systemic chemotherapy, zoledronate does not carry the risk of myelosuppression, infectious complications, or organ toxicity. Its main side-effect profile is an acute-phase reaction with fever and flu-like symptoms, which can be managed with antipyretics, and the risk of hypocalcemia, which can be avoided with appropriate vitamin D levels and calcium supplementation. In our study, all patients were supplemented with oral calcium and had baseline vitamin D levels assessed to be more than 20 µg/L to mitigate the risk of symptomatic hypocalcemia, which was not clinically significant in our cohort. Finally, none of our patients developed osteonecrosis of the jaw, a serious side effect reported in elderly patients using high-dose bisphosphonates in the treatment of multiple myeloma. While zoledronate offers practical advantages, its role appears largely confined to osseous disease. In high-risk multisystem LCH with liver, spleen, or marrow involvement, zoledronate should not replace systemic chemotherapy but may serve as supportive therapy for skeletal symptoms. Nevertheless, its integration as supportive care, especially for skeletal symptoms that persist despite systemic therapy or for sites at high risk of functional compromise, may enhance overall disease management. There is also potential value in its use during the maintenance or post-chemotherapy phases to reduce the burden of residual skeletal disease or to accelerate resolution.

Prospective studies are needed to define optimal dosing, timing relative to systemic therapy, and durability of response. In our cohort, some patients responded to low doses (0.02–0.025 mg/kg) within 1–2 months without recurrence. These observations are hypothesis-generating only, but they suggest a lower-dose, intermittent zoledronate may warrant further study. We propose a regimen of 0.025 mg/kg every 3–4 months (maximum annual dose 0.1 mg/kg), balancing efficacy and safety. This approach should be regarded as a pragmatic institutional practice based on limited retrospective experience rather than a definitive recommended standard. This regimen allows the lowest possible dose to minimise side effects and offer a strategy for rapid pain control should more bony lesions appear, resulting in breakthrough pain. Our center will use this regimen for all patients with LCH who require bisphosphonates, and we hope to report the beneficial outcomes and tolerability of this regimen in future.

Additionally, identifying predictive biomarkers to stratify patients most likely to benefit from bisphosphonate therapy would be valuable. Potential biomarkers may include indicators of systemic disease burden (e.g., number of involved organs or risk of organ involvement), and inflammatory markers such as thrombocytosis, anemia, an elevated erythrocyte sedimentation rate (ESR), and C-reactive protein (CRP), all of which have been associated with disease activity in LCH (19, 20). While our study was not powered to identify predictive biomarkers or definitive response factors, the heterogeneity within the cohort offers some preliminary clinical signals. Patients with localized, symptomatic bone lesions, especially painful lesions in weight-bearing sites, appeared to experience the clearest benefit, with rapid pain relief and subsequent radiographic healing. By contrast, responses were more heterogeneous in patients with multifocal, multiply relapsed, or heavily pretreated disease, in whom zoledronate more often appeared to induce stabilization or partial sclerosis rather than complete lesion resolution. Notably, several of these patients were already relapsing after prior chemotherapy or progressing despite oral therapy when zoledronate was started, suggesting that treatment resistance and disease burden may influence response. For such patients, repeat zoledronate dosing may still provide benefit, but persistent progression may indicate the need for additional systemic therapy or combination approaches.

This study has several important limitations. Limitations include the small sample size, retrospective single-center design, heterogeneous clinical indications for treatment, variation in concurrent or prior systemic therapies, and non-standardized imaging intervals. Because zoledronate was not given within a uniform treatment protocol, it is difficult to isolate its independent contribution from the effects of disease natural history, prior therapy, or concomitant treatment. The cohort also included patients with differing disease extent, relapse status, and treatment goals, which limits direct comparison across cases. In addition, imaging response assessment was based on routine clinical follow-up rather than centrally reviewed, protocolized radiologic evaluation, and long-term skeletal outcomes were not systematically captured. Multicenter studies are needed to validate patient selection criteria, clarify which patients are most likely to benefit, and assess long-term skeletal outcomes.

In conclusion, Intravenous zoledronate appears to be a feasible and generally well-tolerated adjunctive option in the management of pediatric bone LCH. It provides rapid pain relief and radiologic improvement, with minimal toxicity and convenient dosing. These findings support further prospective evaluation but should not be taken as definitive evidence of efficacy. Integration into multimodal treatment strategies for selected patients warrants further prospective evaluation.

## Data Availability

The original contributions presented in the study are included in the article/Supplementary Material, further inquiries can be directed to the corresponding author.

## References

[1] Rodriguez-Galindo C Allen CE. Langerhans cell histiocytosis. Blood. (2020) 135(16):1319–31. 10.1182/blood.2019000934 32106306

[2] Minkov M Steiner M Pötschger U Aricò M Braier J Donadieu J Reactivations in multisystem Langerhans cell histiocytosis: data of the international LCH registry. J Pediatr. (2008) 153(5):700–5.e2. 10.1016/j.jpeds.2008.05.002 18589441

[3] Gadner H Minkov M Grois N Pötschger U Thiem E Aricò M Therapy prolongation improves outcome in multisystem Langerhans cell histiocytosis. Blood. (2013) 121(25):5006–14. 10.1182/blood-2012-09-455774 23589673

[4] Drake MT Clarke BL Khosla S. Bisphosphonates: mechanism of action and role in clinical practice. Mayo Clin Proc. (2008) 83(9):1032–45. 10.4065/83.9.1032 18775204 PMC2667901

[5] Lipton A Small E Saad F Gleason D Gordon D Smith M The new bisphosphonate, Zometa ® (zoledronic acid), decreases skeletal complications in both osteolytic and osteoblastic lesions: a comparison to pamidronate. Cancer Invest. (2002) 20(sup2):45–54. 10.1081/CNV-120014886 12442349

[6] Bowden SA Mahan JD. Zoledronic acid in pediatric metabolic bone disorders. Transl Pediatr. (2017) 6(4):256–68. 10.21037/tp.2017.09.10 29184807 PMC5682380

[7] Elomaa I Blomqvist C Porkka L Holmström T. Experiences of clodronate treatment of multifocal eosinophilic granuloma of bone. J Intern Med. (1989) 225(1):59–61. 10.1111/j.1365-2796.1989.tb00038.x 2918272

[8] Sivendran S Harvey H Lipton A Drabick J. Treatment of Langerhans cell histiocytosis bone lesions with zoledronic acid: a case series. Int J Hematol. (2011) 93(6):782–6. 10.1007/s12185-011-0839-2 21519845

[9] Kudo K Tanaka T Kobayashi A Terui K Ito E. Zoledronic acid for relapsed Langerhans cell histiocytosis with isolated skull bone lesion. Pediatr Int. (2019) 61(3):315–7. 10.1111/ped.13774 30793439

[10] Chellapandian D Makras P Kaltsas G van den Bos C Naccache L Rampal R Bisphosphonates in Langerhans cell histiocytosis: an international retrospective case series. Mediterr J Hematol Infect Dis. (2016) 8(1):1–7. 10.4084/mjhid.2016.033 PMC4928520 27413525

[11] Tsuda H Yamasaki H Tsuji T. Resolution of bone lysis in Langerhans cell histiocytosis by bisphosphonate therapy. Br J Haematol. (2011) 154(3):287. 10.1111/j.1365-2141.2011.08654.x 21595647

[12] Kamizono J Okada Y Shirahata A Tanaka Y. Bisphosphonate induces remission of refractory osteolysis in Langerhans cell histiocytosis. J Bone Miner Res. (2002) 17(11):1926–8. 10.1359/jbmr.2002.17.11.1926 12412797

[13] Takpradit C Vathana N Narkbunnam N Sanpakit K Buaboonnam J. Bisphosphonate therapy for refractory Langerhans cell histiocytosis: a case report. J Med Assoc Thail. (2015) 98(11):1145–9. 26817187

[14] Farran RP Zaretski E Egeler RM. Treatment of Langerhans cell histiocytosis with pamidronate. Am J Pediatr Hematol Oncol. (2001) 23(1):54–6. 10.1097/00043426-200101000-00013 11196272

[15] Morimoto A Shioda Y Imamura T Kanegane H Sato T Kudo K Nationwide survey of bisphosphonate therapy for children with reactivated Langerhans cell histiocytosis in Japan. Pediatr Blood Cancer. (2011) 56(1):110–5. 10.1002/pbc.22703 21108445

[16] Arzoo K Sadeghi S Pullarkat V. Pamidronate for bone pain from osteolytic lesions in Langerhans’-cell histiocytosis. N Engl J Med. (2001) 345(3):225. 10.1056/NEJM200107193450318 11463032

[17] da Costa CET Annels NE Faaij CMJM Forsyth RG Hogendoorn PCW Egeler RM. Presence of osteoclast-like multinucleated giant cells in the bone and nonostotic lesions of Langerhans cell histiocytosis. J Exp Med. (2005) 201(5):687–93. 10.1084/jem.20041785 15753204 PMC2212837

[18] Abildgaard N Rungby J Glerup H Brixen K Kassem M Brincker H Long-term oral pamidronate treatment inhibits osteoclastic bone resorption and bone turnover without affecting osteoblastic function in multiple myeloma. Eur J Haematol. (1998) 61(2):128–34. 10.1111/j.1600-0609.1998.tb01073.x 9714526

[19] Calming U Henter J-I. Elevated erythrocyte sedimentation rate and thrombocytosis as possible indicators of active disease in Langerhans’ cell histiocytosis. Acta Paediatr. (1998) 87(10):1085–7. 10.1080/080352598750031437 9825978

[20] Oh B Lee S Ke Y Kimpo M Yeoh A Quah TC. A “wait-and-see” approach to quiescent single-system Langerhans cell histiocytosis to spare children from chemotherapy. Front Pediatr. (2020) 8:466. 10.3389/fped.2020.00466 32903429 PMC7434943

