# Supplementary material for: Intravenous zoledronate for pediatric langerhans cell histiocytosis with bone involvement
Source: Front Pediatr. 2026 Apr 29;14:1825076. doi: 10.3389/fped.2026.1825076 (PMC13168001; doi:10.3389/fped.2026.1825076)
Supplement: Supplementary file 1 [file Supplementaryfile1.docx]

Supplemental Information for cases 1 to 8:

**Case 1**

A Singaporean Malay girl was diagnosed at 15 months with high-risk multisystem LCH involving the liver, spleen, and lymph nodes. She initially responded to LCH-III chemotherapy but had multiple relapses requiring repeated re-induction and an additional year of systemic therapy. Oral maintenance (prednisolone, 6-mercaptopurine, methotrexate) was attempted but poorly tolerated because of nausea and gastritis.

At 8 years 3 months, she presented with daily right knee pain. Her treatment was limited to oral 6-merceptopurine and oral methotrexate 2 years prior to this presentation. Radiographs showed a distal femoral lytic lesion and multiple calvarial lucencies (Supplemental Figure 1A). Decision was made for IV zoledronate given poor tolerance oral chemotherapy and painful symptomatic lesions. A single IV zoledronate dose of 0.02 mg/kg led to complete pain resolution within 25 days without adverse events; interval imaging at 2 months demonstrated sclerosis, indicating healing (Supplemental Figure 1B). She subsequently experienced a hepatic flare requiring vemurafenib and prednisolone; however, adherence to vemurafenib was inconsistent. Nonetheless, further improvement in the femoral and skull lesions was noted 8 months after zoledronate (Figure 1C).

At 11 years 2 months, she developed new forehead pain and enlarging skull lesions, again in the context of poor tolerance to vemurafenib. She received two further zoledronate doses (0.02 mg/kg then 0.04 mg/kg) without adverse reactions, and imaging showed interval improvement in skull lucencies. A fourth dose was planned but not given as symptoms had resolved.

**Case 2:**

A 47-month-old Singaporean Chinese boy presented with 2 weeks of progressive limping and severe non-ambulatory pain. Radiographs showed mottled lucencies and periosteal reaction along the left tibial shaft, and bone biopsy confirmed unifocal LCH. He had no prior systemic treatment. IV Zoledronate was considered for him in view of localized painful bony LCH in a weight bearing bone. He received four doses of IV zoledronate (0.02–0.04 mg/kg) at 4-monthly intervals. Vitamin D level prior to treatment was 23.9 µg/L, and ionised calcium was 1.17 mmol/L. Pain improved after the first dose, with resolution of limping and full return of function by 4 weeks. He experienced transient fever and vomiting after the initial infusion, which were managed symptomatically. Serial imaging demonstrated progressive endosteal healing and reduced lucency (Supplemental Figure 2), and no other therapy was required.

**Case 3:**

A 68-month-old Singaporean Chinese girl presented with left leg pain causing nocturnal awakening. Radiographs showed an ill-defined mid-tibial lucency with periosteal reaction (Supplemental Figure 3A), and bone biopsy confirmed unifocal LCH. She had no prior systemic treatment. Intravenous zoledronate was considered in view of weight-bearing bone and pain. At 70 months of age, she received a single IV zoledronate dose of 0.02 mg/kg, with only transient post-infusion fever. Pain resolved, and the lesion stabilized within 11 days. One month later, a scalp lump raised concern for progression, and she was started on 6-mercaptopurine and a short course of prednisolone. Therapy was stopped after 6 months. At 9 months post-zoledronate, imaging showed complete resolution of the tibial lesion (Supplemental Figure 3B)**,** and she has remained in remission.

**Case 4:**

A Vietnamese girl was diagnosed at 14 months with multisystem LCH involving the skin, parietal bone, and pituitary gland (diabetes insipidus), and treated with the LCH-III protocol. Due to persistent skull lesions after one year, she received salvage therapy with vincristine and cytarabine (LCH-IV) followed by oral 6-mercaptopurine and methotrexate maintenance.

At 54 months, she was reviewed at our center for persistent scalp lesions. CT brain performed overseas demonstrated left parietal and temporal lytic lesions and a smaller right temporal lesion; skeletal survey confirmed two ovoid lucencies in the parietal and parasagittal regions She had been on oral 6-mercaptopurine and oral methotrexate for 1 year prior to presentation. In view of the new bony lesions, previous heavy treatment and limited finances, IV zoledronate was considered for her. She received a single dose of IV zoledronate 0.02 mg/kg without adverse reactions. On follow-up four months later, head pain had resolved and repeat CT showed disappearance of the parietal lesion with stability of the remaining smaller skull defects. As she remained asymptomatic, further doses were deferred, and she continues on surveillance.

**Case 5:**

A Vietnamese girl was diagnosed at 13 months with multisystem LCH presenting with anemia, hepatosplenomegaly, scalp and skull lesions, and diabetes insipidus. She was treated with the LCH-III protocol. At 40 months, she relapsed with left thigh and right cheek pain and developed a limp with restricted left hip rotation. Skeletal survey showed a lucent lesion at the left acetabulum–superior pubic ramus junction (Supplemental Figure 4A). Her last systemic chemotherapy was 11 months prior to presentation. IV zoledronate was considered in view of multiple painful lesions and lesions over a weight-bearing bone. She received IV zoledronate 0.02 mg/kg, with transient post-infusion fever, followed by resolution of pain and limp within one week.

At 4-month review, she remained well clinically, but imaging revealed a new proximal femoral lucency with a wide transition zone, while the prior lesion showed patchy sclerosis (Supplemental Figure 4B). A second zoledronate dose (0.02 mg/kg) was administered, along with oral 6-mercaptopurine and methotrexate. She continues under follow-up. Subsequent review at 8 months revealed that the lesion continued to improve with sclerosis and no new lesions had developed, and a third dose of 0.02mg/kg was administered and she is on continued follow up.

**Case 6:**

A Vietnamese girl was diagnosed with multifocal bone LCH at 24 months, initially presenting with left thigh pain and a proximal femoral lesion confirmed by biopsy. She was observed initially as the disease appeared unifocal, but subsequently developed right-hand and back pain. CT in Vietnam demonstrated additional lesions in the hand and at T9, and she completed one year of LCH-III therapy.

At 60 months, she was reviewed at our centre for persistent left thigh pain. She remained ambulant. A skeletal survey demonstrated multiple osseous lesions, including right parietal and vertex skull defects, vertebra plana at T1, a healing T9 compression fracture, right third metacarpal shortening, destruction of the right first proximal phalanx, and small lucencies in the left ischium and medial proximal femur. Last systemic chemotherapy was 8 months prior. IV zoledronate was considered in view of lesions in the weight bearing bones (spine). She received IV zoledronate 0.02 mg/kg, with interval improvement and rim sclerosis of lesions at 4-month follow-up. Shen then received a second infusion (0.025 mg/kg) and is on continued follow-up.

**Case 7:**

A Vietnamese boy was diagnosed with multifocal bone LCH at 24 months, presenting with torticollis and swelling of the left jaw and scalp. PET-CT showed lesions in the skull, left humerus, right iliac region, and T11 vertebra with no additional organ involvement. Biopsy confirmed LCH, and he completed one year of LCH-III therapy with remission.

At 39 months, he relapsed with skull pain, and radiographs showed a 1.8-cm lytic skull lesion. He was started on oral prednisolone, 6-mercaptopurine, and methotrexate for 2 months, but the lesion progressed to 2.2 cm after 2 months. In view of the pain and progression despite oral systemic chemotherapy, we considered IV Zoledronate for him. He then received IV zoledronate 0.02 mg/kg. Interval imaging 4 months later showed a slight increase in size (by 0.2 cm) but new peripheral sclerosis, indicating a healing response. A second dose (0.025 mg/kg) was administered, and there was improvement with resolution of some lesions and a decrease in the size of the other lesions thereafter and a third dose of 0.025mg/kg was administered.

**Case 8:** A Japanese girl was diagnosed with multifocal bone Langerhans Cell Histiocytosis (LCH) at 20 months of age and initially treated with the JPLSG LCH-12 protocol. Over the years, she developed multiple osseous relapses involving the skull, spine, ribs, femur, and tibia, managed with re-induction chemotherapy, pamidronate, local radiotherapy, and later low-intensity vinblastine with prednisolone. She achieved remission on indomethacin between 2023 and mid-2024.

At 13 years old, she presented to our clinic with new left parietal scalp pain and a change in bony contour. Radiographs confirmed new lytic lesions in the skull and left proximal tibia, consistent with relapse. Prior to this, last therapy was with oral indomethacin 1 year prior to presentation. In view of the multiply-relapsed disease and previous refractory disease and wanting to spare her from regular indomethacin, we considered her for IV zoledronate therapy. She received a single dose of intravenous zoledronate at 0.025 mg/kg, which was well tolerated with no adverse reactions. After this, the skull lesion resolved and the other lesions, mainly the left proximal tibial lesion, remained stable and she was given a second dose 0.025mg/kg. She is on continued follow-up.


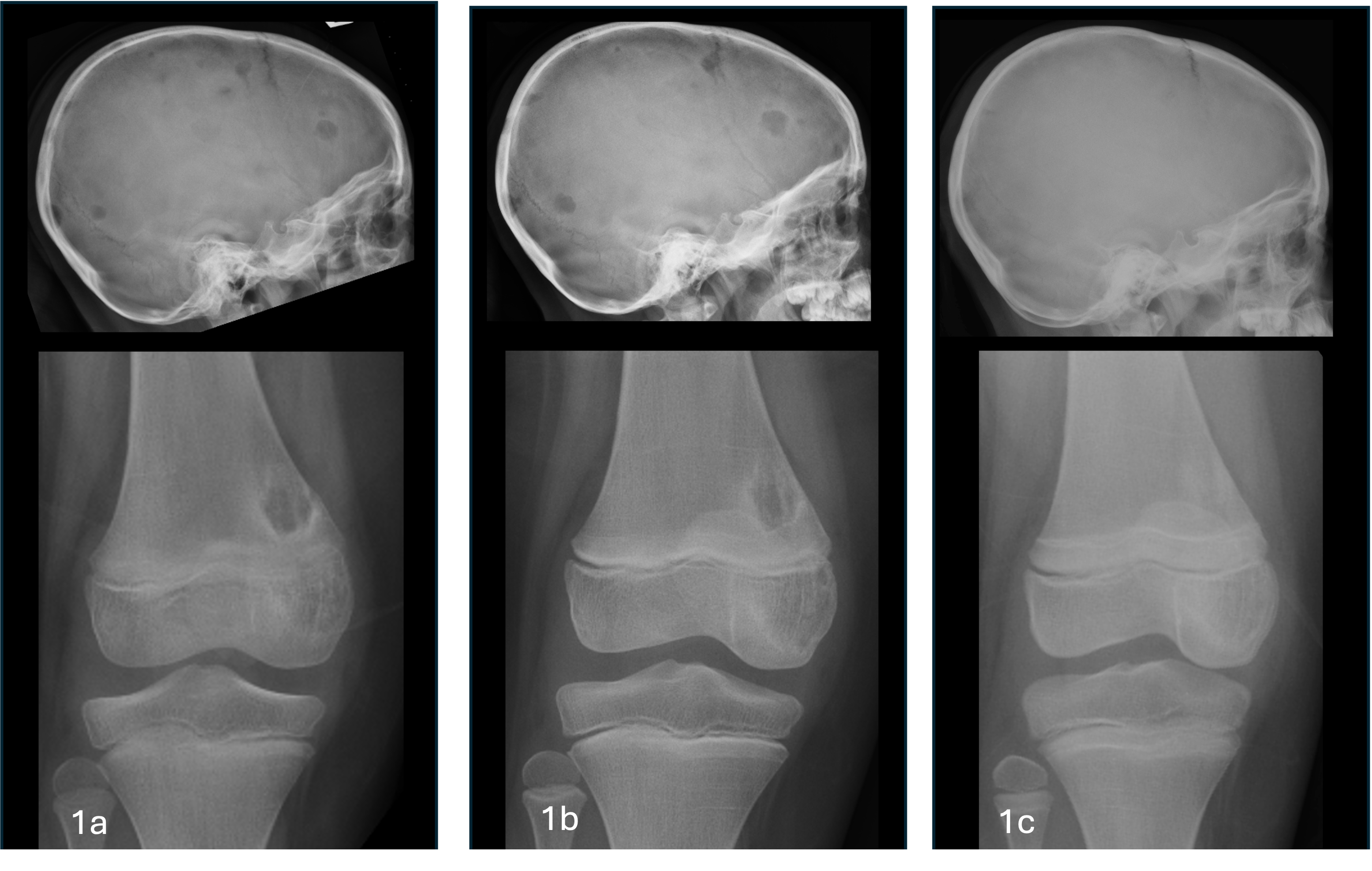


**Supplemental Figure 1.** Improvement of the lesions after IV zoledronate and concomitant oral vemurafenib for liver LCH flare. 1a: Lytic lesion over the distal femur and multiple lucencies over the skull prior to treatment. 1b: Two months after IV zoledronate. 1c: 8 months after IV zoledronate.


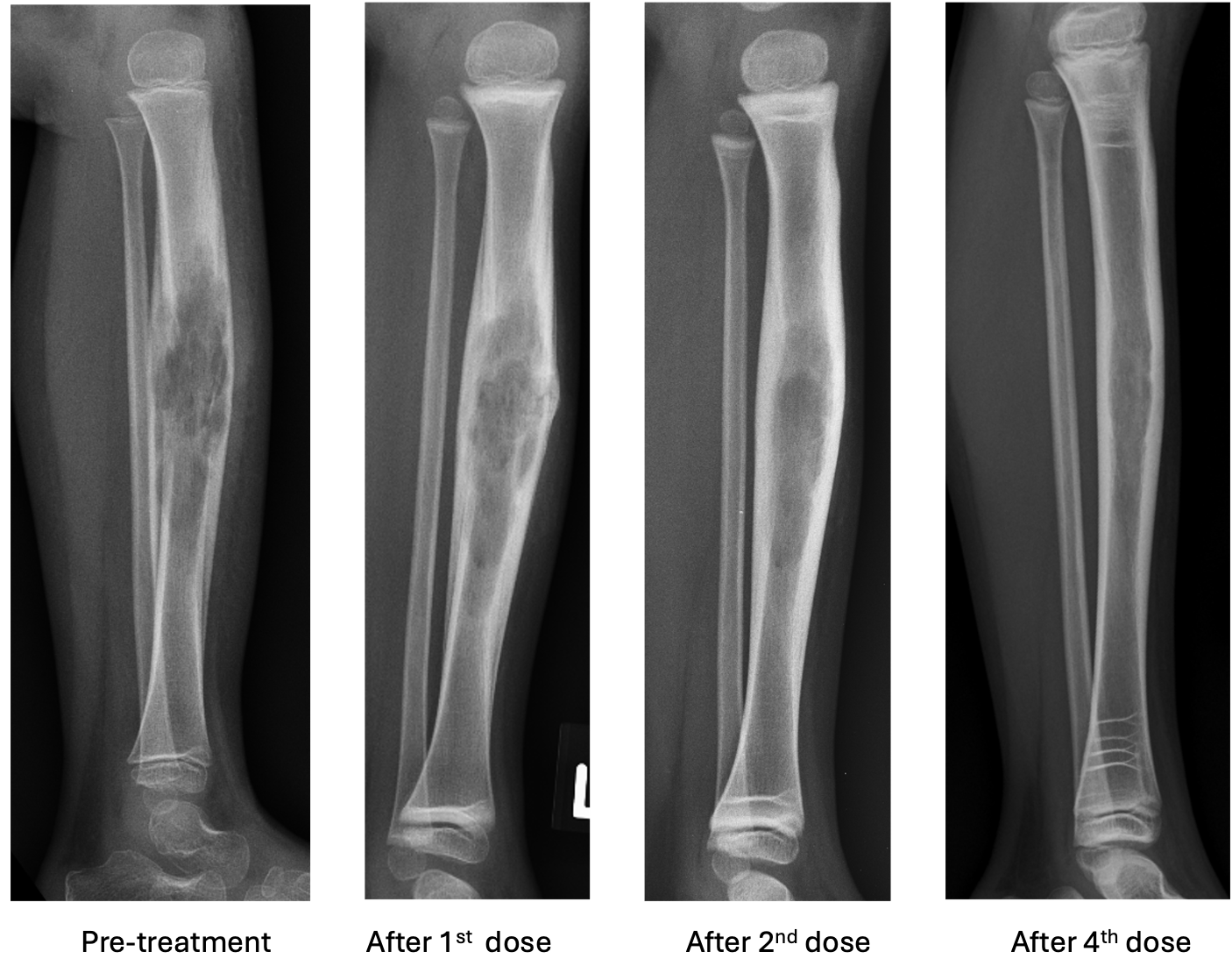


**Supplemental Figure 2.** Imaging throughout treatment, from pre-treatment up to after multiple doses – the resolution of the lucency is appreciated with each subsequent dose.


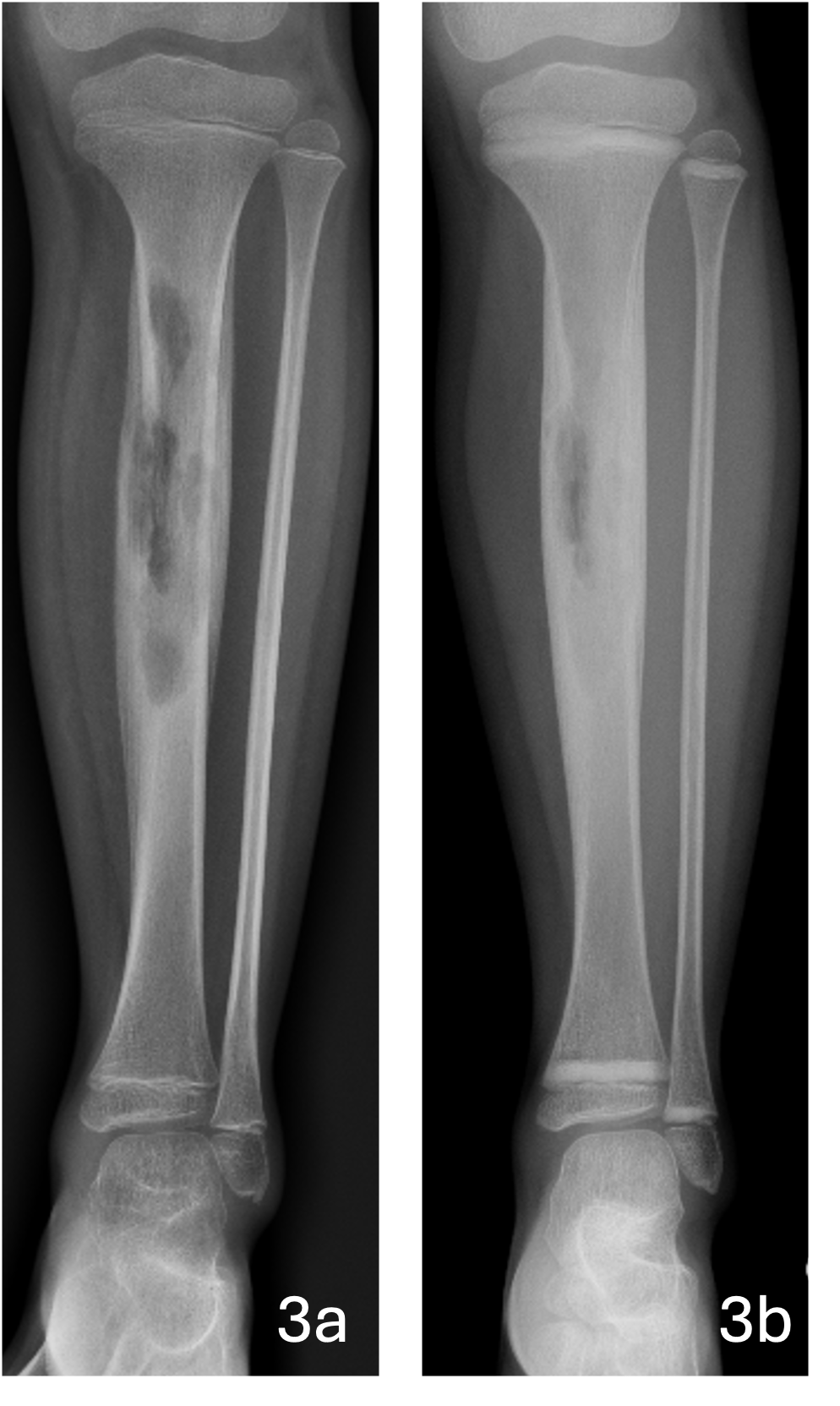


**Supplemental Figure 3.** Before and after images of the left tibia LCH lesion. 3a. Prior to IV zoledronate. 3b. 9 months after IV zoledronate was administered.


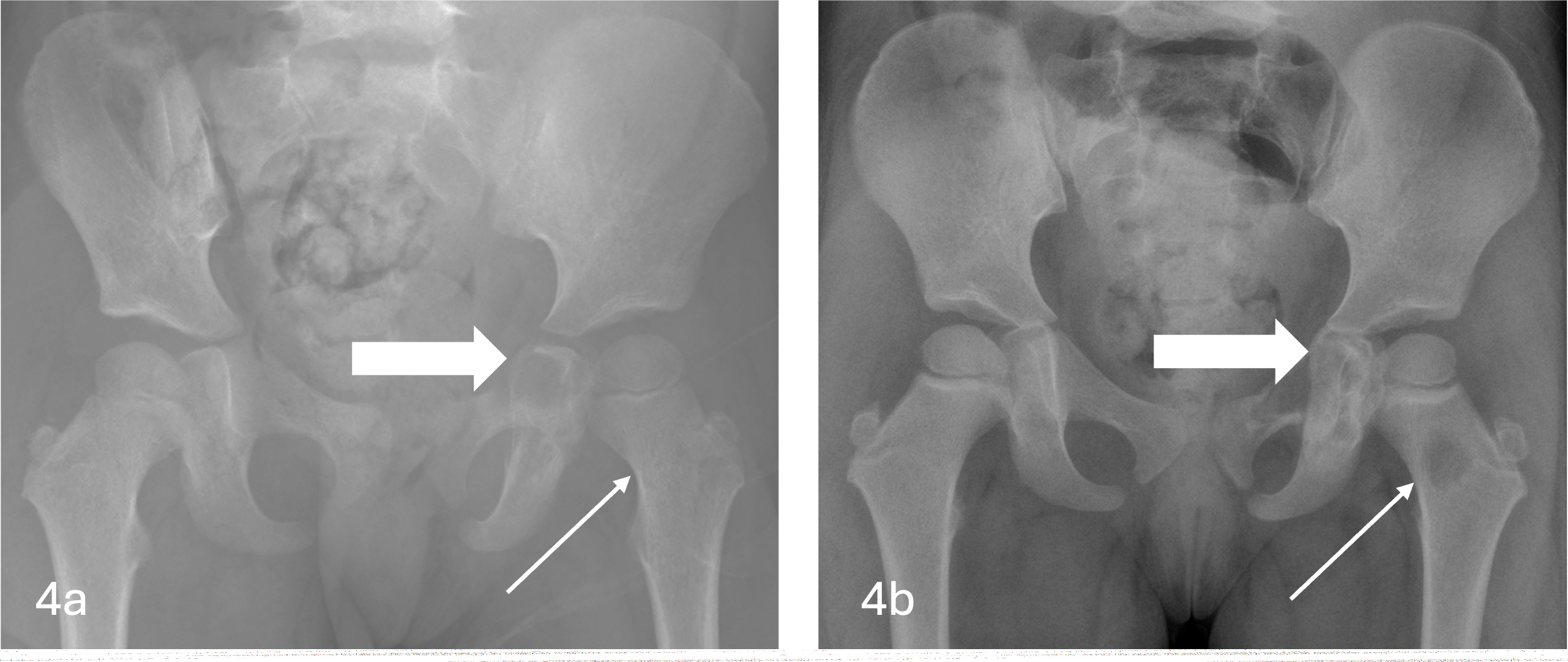


**Supplemental Figure 4**. Before-and-after images of the hip. 4a. Prior to administration of IV zoledronate. The thick white arrow demonstrates the lytic lesion over the left acetabulum. The thin arrow shows the absence of the lytic lesion. 4b 4 months after administration of IV zoledronate. The thick arrow demonstrates the sclerosis and healing of the previous lesion. The thin arrow demonstrates a new lesion in the proximal femur.

Supplemental Table 1. Summary of pediatric patients in the literature with LCH treated with bisphosphonates

| Case | Treatment – dose and duration | Age | Sex | Race | Initial Diagnosis | Lesions at the time of bisphosphonate therapy | Treatment phase | Concomitant Therapy | Side effects | Radiological Response | Time to symptom resolution | Reference |
| --- | --- | --- | --- | --- | --- | --- | --- | --- | --- | --- | --- | --- |
| 1 | iV Zoledronate 2mg/m2 every 4 weeks x 12 cycles | 8Y | F | Not stated | Multifocal bone | Right focal headache with right parietal skull bone resorption and epidual mass | Relapse | PO Celecoxib 100mg daily | Fever, nausea, vomiting, asymptomatic hypocalcemia, hypophosphatemia | Yes, bone lesion and epidual mass disappeared after 12 cycles of chemotherapy | 3 months of treatment | (Kudo et al. 2019) |
| 2 | IV Zoledronate 2mg monthly x 6 times | 10Y | F | Not stated | Multifocal bone | Vertebra plana T7 | Relapse | NSAIDS | None | None reported | 2 months of treatment | (Kikkawa et al. 2013) |
| 3 | PO Alendronate 5mg daily – 2.5 years | 9Y3M | F | Not stated | Multifocal bone | C4 vertebrae, corpus of L3 vertebrae, iliac bones, right pubic ramus | Relapse | Short course of steroids | Mild PTH increase | Yes – 2.5 years. Complete resolution of L3 vertebrae, iliac bones, pubic ramus. C4 lesion treated with bone graft | Not stated | (Chellapandian et al. 2016) |
| 4 | PO Alendronate (dose not stated) x 2 years | 17Y | F | Not stated | Multisystem (RO-) | Occipital bone lesion | Relapse | None | None | Yes – CT resolution after 5 months of treatment | Not stated | (Tsuda, Yamasaki, and Tsuji 2011) |
| 5 | PO Etidronate 200mg/m2/day x 14 days every 3 months x 6 courses | 12M | F | Not stated | Multisystem (RO-) | Skin, cervical lymphadenopathy, osteolytic lesions of the skull | Relapse | None | None | Yes – complete resolution after 6 courses. | Not stated | (Kamizono et al. 2002) |
| 6 | PO Etidronate 200mg/m2/day x 14 days every 3 months x 6 courses | 10M | F | Not stated | Multisystem (RO-) | Skin, cervical lymphadenopathy, osteolytic lesions of the skull | Relapse | None | None | Yes – complete resolution after 6 courses. | Not stated | (Kamizono et al. 2002) |
| 7 | IV pamidronate 1mg/kg x 3 days x 3 months | 4Y2M | M | Not stated | Multifocal bone | Skull, left scapula | Relapse | Not stated | Fever | No – no response after 3 months. | Not stated | (Chellapandian et al. 2016) |
| 8 | IV pamidronate 1mg/kg x 1 day x 6 months | 6Y1M | M | Not stated | Multifocal bone | Skull, left 6^th^ rib, L2 vertebrae | Relapse | Not stated | None | Yes – 6 months. Complete resolution of all lesions | Not stated | (Chellapandian et al. 2016) |
| (continued) | | | | | | | | | | | | |
| Case | Treatment – dose and duration | Age | Sex | Race | Initial Diagnosis | Lesions at the time of bisphosphonate therapy | Treatment phase | Concomitant Therapy | Side effects | Radiological Response | Time to symptom resolution | Reference |
| 9 | IV pamidronate 1mg/kg x 1 day every month x 6 months | 7M | F | Thai | Mutlsystem (RO+) | Anaemia, hepatosplenomegaly, lymphadenopathy, multiple osteolytic lesions. Fracture of intertrochanteric right femur, left tibia and, left scapula | Relapse | Pulse methylprednisolone | None | Yes 6 months, improvement of all lesions. | Not stated | (Takpradit et al. 2015) |
| 10 | IV pamidronate 1mg/kg/day x 3 days monthly x 6 months | 5Y8M | M | Not stated | Multifocal bone | Right mandible, C5 vertebrae | Relapse | Not stated | None | Yes – 6 months. Remodeling and thick periosteal reaction of mandibular lesion, stable at C5 lesion. | Not stated | (Chellapandian et al. 2016) |
| 11 | IV pamidronate 1mg/kg/day x 3 days monthly x 4 months | 2Y8M | M | Not stated | Multisystem (RO-) | Skull, facial bones, scalp, skin | Relapse | Not stated | None | Yes – 4 months – stabilisation of skull and facial bone lesions, reduction of skin and soft tissue lesions | Not stated | (Chellapandian et al. 2016) |
| 12 | IV pamidronate 2mg/kg x 3 days every month x 4 courses | 14Y | M | Not stated | Multisystem, CNS | Panhypopituitarism, diplopia, bilateral optic nerve involvement, diffuse leukoencephalopathy, dysarthria, gait ataxia. Bone – skull, jaw, cervical and thoracic spine and long bone lesions, bone pain. | Relapse | Not stated | None | Yes –after 4 cycles, 4 months after treatment cycle, MRI confirmed regression of zygoma, calvarial lesions. | 1 week after 2^nd^ course | (Peter Farran, Zaretski, and Maarten Egeler 2001) |
| 13 | IV pamidronate 1mg/kg every 4 weeks | 9Y6M | F | Not stated | Not stated | Single bone | Relapse | Meloxicam | None | Yes – no active disease after 2 courses | Not stated | (Morimoto et al. 2011) |
| 14 | IV pamidronate 1mg/kg every 4 weeks | 7Y7M | F | Not stated | Not stated | Multiple bones | Relapse | Methotrexate | None | Yes, partial response in bones | Not stated | (Morimoto et al. 2011) |
| 15 | IV pamidronate 1mg/kg every 4 weeks | 15Y0M | M | Not stated | Not stated | Single bone | Relapse | Meloxicam | None | Yes – no active disease after 2 courses | Not stated | (Morimoto et al. 2011) |
| 16 | IV pamidronate 1mg/kg every 4 weeks | 14Y9M | M | Not stated | Not stated | Multiple bones | Relapse | Meloxicam | None | Yes – no active disease after 2 courses | Not stated | (Morimoto et al. 2011) |
| 17 | IV pamidronate 1-1.25mg/kg x 3 days every 4 weeks | 14Y0M | M | Not stated | Not stated | Bone pain, single bone | Relapse | None | None | Yes – no active disease after 6 courses | Not stated | (Morimoto et al. 2011) |
| (continued) | | | | | | | | | | | | |
| Case | Treatment – dose and duration | Age | Sex | Race | Initial Diagnosis | Lesions at the time of bisphosphonate therapy | Treatment phase | Concomitant Therapy | Side effects | Radiological Response | Time to symptom resolution | Reference |
| 18 | IV pamidronate 1mg/kg every 4 weeks | 13Y7M | F | Not stated | Not stated | Bone pain, single bone | Relapse | None | Fever, fatigue, hypocalcemia | Yes – no active disease after 2 courses | Not stated | (Morimoto et al. 2011) |
| 19 | IV pamidronate 1mg/kg every 4 weeks | 11Y6M | F | Not stated | Not stated | Single bone and soft tissue | Relapse | Vinblastine | Fever, vomiting, diarrhea | No – new bone lesion, soft tissue no active disease | Not stated | (Morimoto et al. 2011) |
| 20 | IV pamidronate 1-1.25mg/kg x 3 days every 4 weeks | 10Y0M | M | Not stated | Not stated | Single bone and bone pain | Relapse | 6MP | None | Yes – no active disease after 4 courses | Not stated | (Morimoto et al. 2011) |
| 21 | IV pamidronate 1mg/kg every 4 weeks | 10Y0M | M | Not stated | Not stated | Bones and bone pain, soft tissue, diabetes insipidus, neurodegeneration, | Relapse | Meloxicam | None | No – no response | Not stated | (Morimoto et al. 2011) |
| 22 | IV pamidronate 1mg/kg every 4 weeks | 4Y8M | M | Not stated | Not stated | Single bone | Relapse | Meloxicam | Uveitis | Yes – no active disease after 2 courses | Not stated | (Morimoto et al. 2011) |
| 23 | IV pamidronate 1mg/kg every 4 weeks | 8Y8M | M | Not stated | Not stated | Bones and bone pain with soft tissue, diabetes insipidus | Relapse | Meloxicam | Hypocalcemia | Yes - no active disease after 5 courses | Not stated | (Morimoto et al. 2011) |
| 24 | IV pamidronate 1mg/kg every 4 weeks | 3Y0M | F | Not stated | Not stated | Bones, skin, DI | Relapse | None | None | Yes – no active disease after 2 courses | Not stated | (Morimoto et al. 2011) |
| 25 | IV pamidronate 1mg/kg every 4 weeks | 2Y3M | F | Not stated | Not stated | Bones, skin | Relapse | Meloxicam | None | Yes – no active disease after 3 courses | Not stated | (Morimoto et al. 2011) |
| 26 | IV pamidronate 1-1.25mg/kg x 3 days every 4 weeks | 8Y0M | M | Not stated | Not stated | Bone with soft tissue | Relapse | Dexamethasone | None | Yes – no active disease after 4 courses | Not stated | (Morimoto et al. 2011) |
| 27 | IV pamidronate 1mg/kg every 4 weeks | 2Y10M | M | Not stated | Not stated | Bones | Relapse | Meloxicam, Prednisolone | None | No – no response | Not stated | (Morimoto et al. 2011) |
| 28 | IV pamidronate 1-1.25mg/kg x 3 days every 4 weeks | 2Y10M | M | Not stated | Not stated | Bones with soft tissue | Relapse | None | None | Yes – no active disease after 2 courses | Not stated | (Morimoto et al. 2011) |
